# Hierarchical Discriminant Analysis

**DOI:** 10.3390/s18010279

**Published:** 2018-01-18

**Authors:** Di Lu, Chuntao Ding, Jinliang Xu, Shangguang Wang

**Affiliations:** State Key Laboratory of Networking and Switching Technology, Beijing University of Posts and Telecommunications, Beijing 100876, China; ludi8418@gmail.com (D.L.); jlxu@bupt.edu.cn (J.X.); sgwang@bupt.edu.cn (S.W.)

**Keywords:** Internet of Things, intelligent data, subspace learning, marginal fisher analysis, dimensionality reduction, discriminant neighborhood embedding

## Abstract

The Internet of Things (IoT) generates lots of high-dimensional sensor intelligent data. The processing of high-dimensional data (e.g., data visualization and data classification) is very difficult, so it requires excellent subspace learning algorithms to learn a latent subspace to preserve the intrinsic structure of the high-dimensional data, and abandon the least useful information in the subsequent processing. In this context, many subspace learning algorithms have been presented. However, in the process of transforming the high-dimensional data into the low-dimensional space, the huge difference between the sum of inter-class distance and the sum of intra-class distance for distinct data may cause a bias problem. That means that the impact of intra-class distance is overwhelmed. To address this problem, we propose a novel algorithm called Hierarchical Discriminant Analysis (HDA). It minimizes the sum of intra-class distance first, and then maximizes the sum of inter-class distance. This proposed method balances the bias from the inter-class and that from the intra-class to achieve better performance. Extensive experiments are conducted on several benchmark face datasets. The results reveal that HDA obtains better performance than other dimensionality reduction algorithms.

## 1. Introduction

In recent years, the high penetration rate of Internet of Things (IoT) in all activities of everyday life is fostering the belief that for any kind of high-dimensional IoT data there is always a solution able to successfully deal with it. The proposed solution is the dimensionality reduction algorithm. It is well known that the high dimensionality of feature vectors is a critical problem in practical pattern recognition. Naturally, dimensionality reduction technology has been shown to be of great importance to data preprocessing, such as face recognition [1] and image retrieval [2]. It reduces the computational complexity through reducing the dimension and improving the performance at the same time. A general framework [3] for dimensionality reduction defines a general process according to describing the different purposes of the dimensionality reduction algorithm that was proposed. Among this general framework, the dimensionality reduction is divided into unsupervised algorithms and supervised algorithms.

Unsupervised algorithms conduct datasets without labels, including principle component analysis (PCA) [4], locality preserving projection (LPP) [5], neighborhood preserving embedding (NPE) [6], etc. Supervised algorithms conduct datasets with labels that aim to present better performance and low complexity. Linear discriminant analysis (LDA), local discriminant embedding (LDE) [7], discriminant sparse neighborhood preserving embedding (DSNPE) [8], regularized coplanar discriminant analysis (RCDA) [9], marginal Fisher analysis (MFA) [3,5,10], discriminant neighborhood embedding (DNE) [11], locality-based discriminant neighborhood embedding(LDNE) [12], and double adjacency graphs-based discriminant neighborhood embedding (DAG-DNE) [13] are typical supervised algorithms.

The unsupervised algorithm PCA [4] adopts linear transformation to achieve dimensional reduction commendably. However, PCA shows a noneffective performance in handling manifold data. Then, LLE [14] is proposed, which firstly uses linear coefficients to represent the local geometry of a given point, then explores a low-dimensional embedding for reconstruction in the subspace. However, LLE only defines mappings on the training data which means it is ill-defined in defining mappings on the testing data. To remedy this problem, NPE [6], orthogonal neighborhood preserving projection (ONPP) [15] and LPP are put on the table. They figure out the problem of LLE while preserving the original structure. NPE and ONPP solve the generalized eigenvalue problem with different constraint conditions. LPP [5] reserves the local structure by preferring an embedding algorithm. This algorithm extends to new points easily. However, as an unsupervised dimensionality reduction algorithm, LPP only shows good performance in datasets without labels.

To address the problem that unsupervised algorithms cannot work well in classification tasks, many supervised algorithms are proposed, such as linear discriminant analysis (LDA) [16], which maximizes the inter-class scatter, minimizes the intra-class scatter simultaneously and finds appropriate project directions for classification tasks. However, LDA still has some limitations. For instance, LDA only considers the global Euclidean structure. Marginal Fisher analysis [10] is proposed as an improved algorithm to surmount this problem. MFA constructs the penalty graph and the intrinsic graph to hold the local structure thereby solving the limitation problem of LDA. Both LDA and MFA can discover the projection directions that simultaneously maximize the inter-class scatter and minimize the intra-class scatter. DAG-DNE compensates for the small inter-class scatter in the subspace of DNE [11]. DAG-DNE constructs a homogeneous neighbor graph and heterogeneous neighbor graph to maintain the original structure perfectly. It maximizes the margin between the inter-class scatter and intra-class scatter so that points in the same class are compact and points in the different classes become separable in the subspace at the same time. However, DAG-DNE and all above dimensionality reduction algorithms, optimize intra-class and inter-class simultaneously. The inter-class scatter is larger than the intra-class scatter, thus it will aim at inter-class but ignore the intra-class. Thus, the huge difference between the sum of inter-class distance and the sum of intra-class distance for distinct datasets may cause a bias problem, which means the impact of intra-class distance is tiny in optimization tasks. Naturally, the separation optimization idea comes to mind.

In this paper, we proposed a novel supervised subspace learning algorithm to further increase the performance, called hierarchical discriminant analysis. More concretely, we construct two adjacency graphs by distinguishing homogeneous and heterogeneous neighbors in HDA. Then HDA optimizes inter-class distance and intra-class distance independently. We minimize the intra-class distance first, then maximize the inter-class distance. Because of the hierarchical work, the optimization of intra-class distance and inter-class distance are detached. The process of optimization does not have to be biased to the inter-class scatter. Both of them get the best results. Thus, the influence between classes can be eliminated and we can find a good projection matrix.

The rest of this paper is structured as follows. In Section 2 we introduce the background on MFA, LDNE and DAG-DNE.I. Section 3, we dedicate to introducing HDA and revealing its connections with MFA, LDNE and DAG-DNE. In Section 4, several simulation experiments applying our algorithm are presented to verify its effectiveness. This is followed by the conclusions in Section 5.

## 2. Backgroud

In this section we emphatically introduce three classical algorithms, MFA [10], LDNE [12] and DAG-DNE [13], which are related to our research point.

### 2.1. Marginal Fisher Analysis

MFA proposes a new criteria. It can conquer the limitation of LDA by characterizing the intra-class compactness and inter-class separability. MFA algorithm follows the steps below:

(1) Construct the adjacency matrices. There are intrinsic graph characterizing the intra-class compactness and penalty graph characterizing the inter-class separability. For each sample $x_{i}$, the intra-class matrix $S^{w}$ is defined as:
  (1) $$S_{ij}^{w}=\left\{\begin{array}{c} 1,\;x_{i}\in N_{K_{1}}\left(x_{j}\right)\;\mathrm{or}\;x_{j}\in N_{K_{1}}\left(x_{i}\right)\\ 0,\;\;\mathrm{otherwise} \end{array}\right.$$
  where $N_{K_{1}}\left(x_{i}\right)$ represents the index set of the $K_{1}$ nearest neighbors of node $x_{i}$ in the same class.
  For each sample $x_{i}$, the inter-class matrix $S^{b}$ is defined as:
  (2) $$S_{ij}^{b}=\left\{\begin{array}{c} 1,\;x_{i}\in P_{K_{2}}\left(x_{j}\right)\;\mathrm{or}\;x_{j}\in P_{K_{2}}\left(x_{i}\right)\\ 0,\;\;\mathrm{otherwise} \end{array}\right.$$
  where $P_{K_{2}}\left(x_{i}\right)$ expresses the set of the $K_{2}$ nearest neighbors of point $x_{i}$ that are in the different classes.
(2) Marginal Fisher Analysis Criterion. Find the optimal projection direction by minimizing the intra-class compactness and maximizing the inter-class separability at the same time.
  (3) $$\min_{P}\frac{tr\left(P^{T}X\left(D^{w}-S^{w}\right)X^{T}P\right)}{tr\left(P^{T}X\left(D^{b}-S^{b}\right)X^{T}P\right)}$$
  tr(·) is the trace of a matrix, $D_{ii}^{w}=\sum_{j}S_{ij}^{w},D_{ii}^{b}=\sum_{j}S_{ij}^{b}$, and P is composed of the optimal *r* projection vectors, X is the set of samples.

### 2.2. Locality-Based Discriminant Neighborhood Embedding

LDNE uses a weight function instead of 1 or 0 to adopt the adjacency graph. It maximizes the difference between the inter-class scatter and the intra-class scatter to capture the top projection matrix. The LDNE algorithm is done by the following steps:

(1) Use K-nearest neighbors to construct the adjacency. The adjacency weight matrix S is declared as:
  (4) $$S_{ij}=\left\{\begin{array}{cc} -\exp\left(\frac{-\left|\right|x_{i}-x_{j}|{|}^{2}}{\beta }\right), & x_{i}\in S_{K}^{w}\left(x_{j}\right)\;\mathrm{or}\;x_{j}\in S_{K}^{w}\left(x_{i}\right)\\ +\exp\left(\frac{-\left|\right|x_{i}-x_{j}|{|}^{2}}{\beta }\right), & x_{i}\in S_{K}^{b}\left(x_{j}\right)\;\mathrm{or}\;x_{j}\in S_{K}^{b}\left(x_{i}\right)\\ \;\;\;0, & \mathrm{otherwise} \end{array}\right.$$
  where $S^{\omega }\left(x_{i}\right)$ denotes the intra-class neighbors of each sample point $x_{i}$, $S^{b}\left(x_{i}\right)$ denotes the inter-class neighbors of $x_{i}$, and the parameter β is a regulator.
(2) Feature mapping: Optimize the following objective function:
  (5) $$\left\{\begin{array}{c} \max_{P}\;\mathrm{tr}\{P^{T}\mathrm{XH}X^{T}P\}\\ \;s.t.\;\;\;P^{T}P=I \end{array}\right.$$
  where H=D−S, and D is a diagonal matrix with $D_{ii}=\sum_{j}S_{ij}$. Function (5) can be considered as a generalized eigen-decomposition problem:
  (6) $$\mathrm{XH}X^{T}P=\lambda P$$
  The optimal projection P consists of *r* eigenvectors corresponding to the *r* largest eigenvalues.

### 2.3. Double Adjacency Graphs-Based Discriminant Neighborhood Embedding

DAG-DNE is a linear manifold learning algorithm based on DNE, which constructs homogeneous and heterogeneous neighbor adjacency graphs. This algorithm follows the steps below:

(1) Construct two adjacency graphs. Let $A^{w}$ and $A^{b}$ be the intra-class and inter-class adjacency matrices. $x_{i}$ is the sample point. The intra-class adjacency matrix $A^{w}$ is defined as
  (7) $$A_{ij}^{w}=\left\{\begin{array}{c} 1,\;x_{i}\in S_{K}^{w}\left(x_{j}\right)\;\mathrm{or}\;x_{j}\in S_{K}^{w}\left(x_{i}\right)\\ 0,\;\;\mathrm{otherwise} \end{array}\right.$$
  The inter-class adjacency matrix $A^{b}$ is defined as
  (8) $$A_{ij}^{b}=\left\{\begin{array}{c} 1,\;x_{i}\in S_{K}^{b}\left(x_{j}\right)\;\mathrm{or}\;x_{j}\in S_{K}^{b}\left(x_{i}\right)\\ 0,\;\;\mathrm{otherwise} \end{array}\right.$$
(2) Optimize the following objective function by finding a projection P.
  (9) $$\left\{\begin{array}{c} \max_{P}\;\mathrm{tr}\{P^{T}\mathrm{XG}X^{T}P\}\\ \;s.t.\;\;\;P^{T}P=I \end{array}\right.$$
  where $G=D^{b}-A^{b}-D^{w}+A^{w}$, and $D^{b}$ and $D^{w}$ are diagonal matrices with $D_{ii}^{b}=\sum_{j}A_{ij}^{b}$ and $D_{ii}^{w}=\sum_{j}A_{ij}^{w}$. The projection matrix P can be solved through the generalized eigenvalue problem as follows:
  (10) $$\mathrm{XG}X^{T}P=\lambda P$$
  The optimal projection P consists of *r* eigenvectors corresponding to the *r* largest eigenvalues.

From optimizing the equation of the related work we can see that the optimization work is achieved through maximizing the distance of inter-class minus the distance of the intra-class or maximizing the distance inter-class divided by the intra-class. Thus, the inter-class and intra-class are simultaneously optimized. Our hierarchical discriminant analysis separates the inter-class and the intra-class. This method avoids the interference between the inter-class and the intra-class. The detail of our algorithm will be presented in the next part.

## 3. Hierarchical Discriminant Analysis

This section discusses a novel dimensional reduction algorithm called hierarchical discriminant analysis. In MFA and DAG-DNE, two adjacency matrices for a node’s homogenous neighbors and heterogeneous neighbors are optimized simultaneously. The huge difference between the sum of inter-class distance and the sum of intra-class distance for distinct data may cause a bias problem, which is too weak for intra-class scatter to play a fundamental role. HDA considers the adjacency graphs respectively, which optimizes the sum of distances between each node and its neighbors of the same class firstly, and then optimizes the sum of distances between the nodes of different classes.

### 3.1. HDA

Suppose ${\left\{\left(x_{i},y_{i}\right)\right\}}_{i=1}^{N}$ is the set of training points, where *N* is the number of training points, $x_{i}\in R^{D},y_{i}\in \left\{1,2,\ldots ,c\right\}$, $y_{i}$ is the class label of $x_{i}$, *d* is the dimensionality of points, and *c* is classes’ number. For these unclassified training sets, we come up with a potential manifold subspace. We use matrix transformation to project the original high-dimensional data into a low-dimensional space. By doing so, homogeneous samples are compacted and the heterogeneous samples are scattered in low-dimensional data. Consequently, the computational complexity and classification performance are improved.

Similar to DAG-DNE [13], HDA constructs two adjacency matrices respectively, the intra-class and inter-class adjacency matrices. Given a sample $x_{i}$, we suppose the set of its *k* homogenous and heterogeneous neighbors are $\pi_{k}^{+}\left(x_{i}\right)$ and $\pi_{k}^{-}\left(x_{i}\right)$. We construct the intra-class adjacency matrix $F^{w}$ and the inter-class adjacency matrix $F^{b}$.

(11) $$F_{ij}^{w}=\left\{\begin{array}{cc} +1, & x_{i}\in \pi_{k}^{+}\left(x_{j}\right)\;\mathrm{or}\;x_{j}\in \pi_{k}^{+}\left(x_{i}\right)\\ 0 & \mathrm{otherwise} \end{array}\right.$$

(12) $$F_{ij}^{b}=\left\{\begin{array}{cc} +1, & x_{i}\in \pi_{k}^{-}\left(x_{j}\right)\;\mathrm{or}\;x_{j}\in \pi_{k}^{-}\left(x_{i}\right)\\ 0 & \mathrm{otherwise} \end{array}\right.$$

And the local intra-class scatter is defined as:

(13) $$\begin{array}{cc} \Phi \left(P_{1}\right) & =\sum_{x_{i}\in \pi_{k}^{+}\left(x_{j}\right)}\sum_{x_{j}\in \pi_{k}^{+}\left(x_{i}\right)}{∥{P_{1}}^{T}x_{i}-{P_{1}}^{T}x_{j}∥}^{2}F_{ij}^{w}\\ & =\sum_{x_{i}\in \pi_{k}^{+}\left(x_{j}\right)}\sum_{x_{j}\in \pi_{k}^{+}\left(x_{i}\right)}\left\{{P_{1}}^{T}\left(x_{i}-x_{j}\right){\left(x_{i}-x_{j}\right)}^{T}P_{1}\right\}F_{ij}^{w}\\ & =\sum_{x_{i}\in \pi_{k}^{+}\left(x_{j}\right)}\sum_{x_{j}\in \pi_{k}^{+}\left(x_{i}\right)}\left\{{P_{1}}^{T}x_{i}{x_{i}}^{T}P_{1}-{P_{1}}^{T}x_{i}{x_{j}}^{T}P_{1}-{P_{1}}^{T}x_{j}{x_{i}}^{T}P_{1}+{P_{1}}^{T}x_{j}{x_{j}}^{T}P_{1}\right\}\\ & =2\sum_{x_{i}\in \pi_{k}^{+}\left(x_{j}\right)}\left\{{P_{1}}^{T}x_{i}{x_{i}}^{T}P_{1}\right\}D_{ii}^{w}-2\sum_{x_{i}\in \pi_{k}^{+}\left(x_{j}\right)}\sum_{x_{j}\in \pi_{k}^{+}\left(x_{i}\right)}\left\{{P_{1}}^{T}x_{i}{x_{j}}^{T}P_{1}\right\}F_{ij}^{w}\\ & =2\{\left\{{P_{1}}^{T}\left(\sum_{x_{i}\in \pi_{k}^{+}\left(x_{j}\right)}x_{i}D_{ii}^{w}{x_{i}}^{T}\right)P_{1}\right\}-\left\{{P_{1}}^{T}\left(\sum_{x_{i}\in \pi_{k}^{+}\left(x_{j}\right)}\sum_{x_{j}\in \pi_{k}^{+}\left(x_{i}\right)}x_{i}F_{ij}^{w}{x_{j}}^{T}\right)P_{1}\right\}\}\\ & =2{P_{1}}^{T}X\left(D^{w}-F^{w}\right)X^{T}P_{1} \end{array}$$

where $D^{w}$ is a diagonal matrix and its entries are column sum of $F^{w}$, i.e., $D_{ii}^{w}=\sum_{j}F_{ij}^{w}$. First, we minimize the intra-class scatter, which means:

(14) $$\left\{\begin{array}{c} \min\Phi \left(P_{1}\right)\\ s.t.{P_{1}}^{T}P_{1}=I \end{array}\right.$$

The objective function can be rewritten by some algebraic steps:

(15) $$\begin{array}{cc} \Phi \left(P_{1}\right) & =2tr\left\{{P_{1}}^{T}X\left(D^{w}-F^{w}\right)X^{T}P_{1}\right\}\\ & =2tr\left\{{P_{1}}^{T}\mathrm{XS}X^{T}P_{1}\right\} \end{array}$$

where $S=D^{w}-F^{w}$. Therefore, the form of trace optimization function can be rewritten as

(16) $$\left\{\begin{array}{c} \min_{P_{1}}\mathrm{tr}\left\{{P_{1}}^{T}\mathrm{XS}X^{T}P_{1}\right\}\\ s.t.{P_{1}}^{T}P_{1}=I \end{array}\right.$$

While the local inter-class scatter is defined as:

(17) $$\begin{array}{cc} \Psi \left(P\right) & =\sum_{x_{i}\in \pi_{k}^{-}\left(x_{j}\right)}\sum_{x_{j}\in \pi_{k}^{-}\left(x_{i}\right)}{∥P^{T}x_{i}-P^{T}x_{j}∥}^{2}\\ & =2P^{T}X\left(D^{b}-F^{b}\right)X^{T}P \end{array}$$

where $D^{b}$ is a diagonal matrix and its entries are the column sum of $F^{b}$, i.e., $D_{ii}^{b}=\sum_{j}F_{ij}^{b}$.

Now, we maximize the inter-class scatter:

(18) $$\left\{\begin{array}{c} \max\Psi \left(P\right)\\ s.t.P^{T}P=I \end{array}\right.$$

It is worth noting that the training set has changed since first minimization, and we rewrite this function as:

(19) $$\begin{array}{cc} \Psi \left(P\right) & =2tr\left\{P^{T}X_{new}\left(D^{b}-F^{b}\right)X_{new}^{T}P\right\}\\ & =2tr\left\{P^{T}X_{new}MX_{new}^{T}P\right\} \end{array}$$

where $X_{new}=XP_{1}$.

So that the optimization problem as shown below:

(20) $$\left\{\begin{array}{c} \max_{P}\mathrm{tr}\left\{P^{T}X_{new}{\mathrm{MX}}_{new}^{T}P\right\}\\ s.t.P^{T}P=I \end{array}\right.$$

Since S and M are real symmetric matrices, the optimization scatters (17) and (20) are same as the eigen-decomposition problem of the matrices $\mathrm{XS}X^{T}$ and $\mathrm{XM}X^{T}$. The two projection matrices P are composed of the egienvetors that corresponding to the egienvalues of $\mathrm{XS}X^{T}$ and $\mathrm{XM}X^{T}$. The optimal solutions $P_{1}$ and P have the form $P=\left[p_{1},\ldots ,p_{r}\right]$.

Therefore, the image of any point $x_{i}$ can be represented as $v_{i}=P^{T}x_{i}$. The details about HDA are given below (Algorithm 1).

**Algorithm 1** Hierarchical Discriminant Analysis**Input:** A training set ${\left\{\left(x_{i},y_{i}\right)\right\}}_{i=1}^{N}$, and the dimensionality of discriminant subspace *r*. **Output:** Projection matrix P.1:Compute the intra-class adjacency graph $F^{w}$$$F_{ij}^{w}=\left\{\begin{array}{ccc} +1, & x_{i}\in \pi_{k}^{+}\left(x_{j}\right)\;or\;x_{j}\in \pi_{k}^{+}\left(x_{i}\right)0 & otherwise \end{array}\right.$$and the inter-class adjacency matrix $F^{b}$.$$F_{ij}^{b}=\left\{\begin{array}{ccc} +1, & x_{i}\in \pi_{k}^{-}\left(x_{j}\right)\;or\;x_{j}\in \pi_{k}^{-}\left(x_{i}\right)0 & otherwise \end{array}\right.$$2:Minimize the intra-class distance by decomposing the matrix $\mathrm{XS}X^{T}$, where $S=D^{w}-F^{w}$. Let egienvalues be $\lambda_{i}$, i=1,…,d and their corresponding egienvectors be $\lambda_{1}\leq \lambda_{2}\leq \cdots \leq \lambda_{d}$.3:Choose the first *r* smallest egienvalues so that return $P_{1}=\left[p_{1},\ldots ,p_{r}\right]$.4:Compute the new input $X_{new}=XP_{1}$.5:Maximize the inter-class distance by decomposing the matrix $\mathrm{XM}X^{T}$, where $S=D^{b}-F^{b}$. Let egienvalues be $\lambda_{i}$, i=1,…,d and their corresponding egienvectors be $\lambda_{1}\geq \lambda_{2}\geq \cdots \geq \lambda_{d}$.6:Choose the first *r* largest egienvalues so that return $P=\left[p_{1},\ldots ,p_{r}\right]$.


### 3.2. Comparisons with MFA, LDNE and DAG-DNE

HDA, LDNE and DAG-DNE are all dimensionality reduction algorithms. In this section, we will probe into the relationships between HDA and the other three algorithms.

#### 3.2.1. HDA vs. MFA

These two algorithms both build an intrinsic(intra-class) graph and penalty(inter-class) graph to keep the original structure of the given data. MFA designs two graphs to find the projection matrix which characterizes the intra-class closely and inter-class separately. However, MFA chooses the dimensionality of the discriminant subspace by experience, which causes some important information to be lost. HDA maximizes the distance of the inter-class and minimizes the distance of the intra-class hierarchically. It uses this method to find the projection matrix to estimate the dimension of the discriminant subspace.

#### 3.2.2. HDA vs. LDNE

Both HDA and LDNE are supervised subspace learning algorithms and build two adjacency graphs to keep the original structure of the given data. LDNE assigns different weights to intra-class neighbors and inter-class neighbors of a given point. It follows the ‘locality’ idea of LPP, and produces balanced links through the set up connection between each point and its heterogeneous neighbors. By analyzing the experimental result, we get an effectiveness result on the subspace.

#### 3.2.3. HDA vs. DAG-DNE

DAG-DNE algorithm maintains balanced links by constructing two adjacency graphs. Through this method, samples in the same class are compact and samples in different classes are separable in the discriminant subspace. However, DAG-DNE optimizes the heterogeneous neighbors and homogeneous neighbors simultaneously. By doing this, the distance of inter-class scatter is not wide enough in the subspace. The HDA algorithm also constructs two graphs, and separately minimizes the within-class distance first, then maximizes the inter-class distance. Because of the hierarchical work, the optimization of intra-class distance and inter-class distance are detached. The process of optimization is not biased to the inter-class scatter. As a consequence, the intra-class is compact while the inter-class is separated in the subspace.

## 4. Experiments

**Figure 1:**
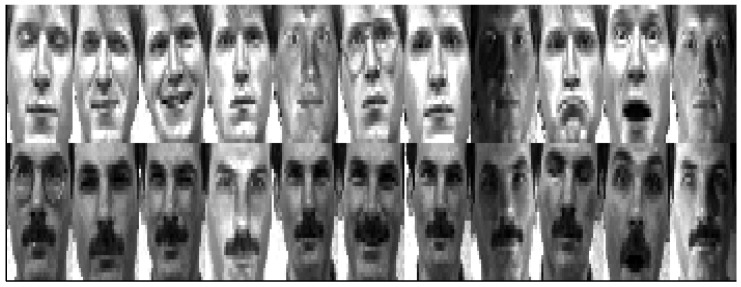
Samples from the Yale face dataset.

**Figure 2:**
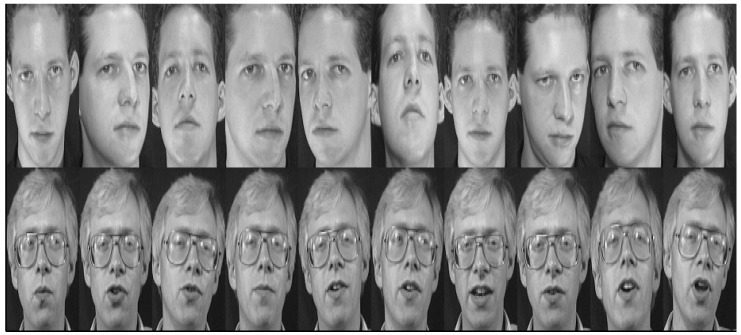
Samples from the ORL face dataset.

**Figure 3:**
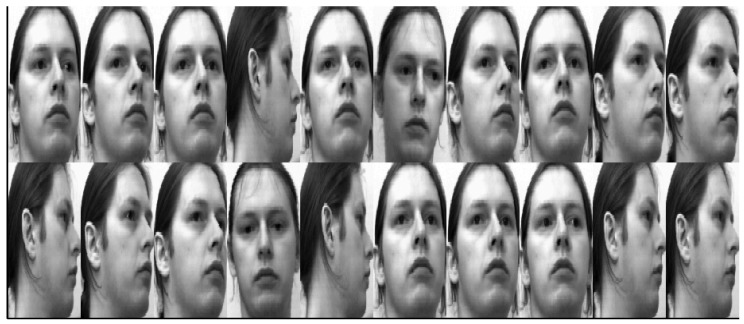
Samples from the UMIST face dataset.

We illustrate a set of experiments to confirm the performance of HDA on image classification in this part. Several benchmark datasets are used, i.e., Yale, Olivetti Research Lab (ORL) and UMIST. The samples from three datasets are shown in Figure 1, Figure 2 and Figure 3. We compare HDA with other representative dimensional reduction algorithms, including LPP, MFA and DAG-DNE.W. Then we choose the nearest neighbor parameter K for those algorithms when constructing the adjacency graphs. We exhibited the results in the form of mean recognition rate.

### 4.1. Yale Dataset

The Yale datset is constructed by the Yale Center for Computational Vision and Control. It contains 165 gray scale images of 15 individuals. These images display variations in lighting condition and facial expression (normal, happy, sad, sleepy, surprised and wink). Each image is 32 × 32 dimension.

**Figure 4:**
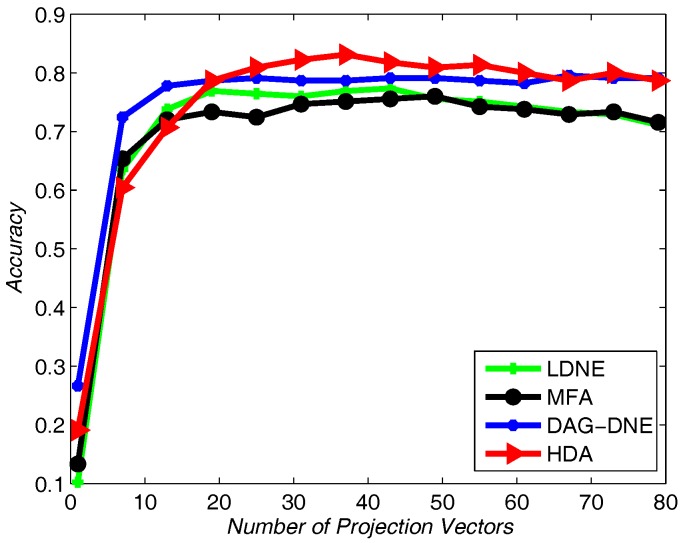
Accuracy vs. Number of Projection Vectors on the Yale dataset under K=1.

**Figure 5:**
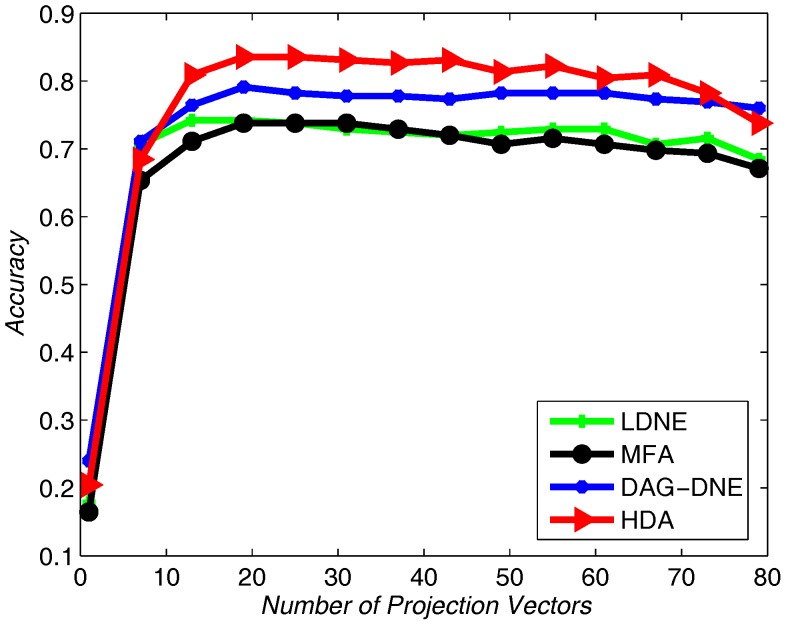
Accuracy vs. Number of Projection Vectors on the Yale dataset under K=3.

**Figure 6:**
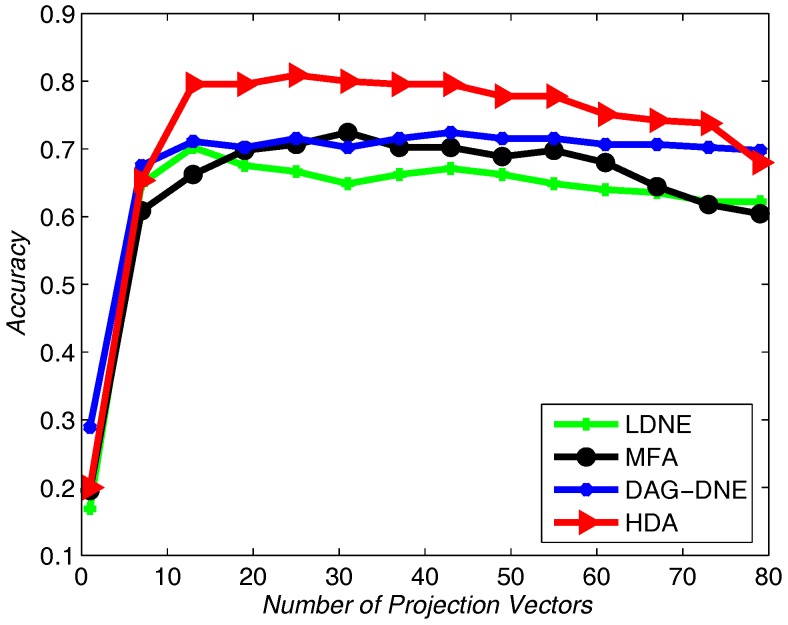
Accuracy vs. Number of Projection Vectors on the Yale dataset under K=5.

We choose 60% from the dataset as training samples, and the rest of the samples for testing. The nearest neighbor parameter *K* can be taken as 1, 3 and 5. Figure 4 shows the average accuracy rate of K=1 after running the four algorithms 10 times. Figure 5 with K=3 and Figure 6 with K=5 also obtain similar results like Figure 4 after being run 10 times. From these three figures, we can see that HDA has a higher accuracy rate than other three algorithms and we can gain the best performance.

### 4.2. UMIST Dataset

The UMIST dataset consists of 564 images of 20 individuals. The dataset takes race, sex and appearance into account. Each individual takes several poses from profile to frontal views. The original size of each image is 112 × 92 pixels. In our experiments, the whole dataset is resized to 32 × 32 pixels.

**Figure 7:**
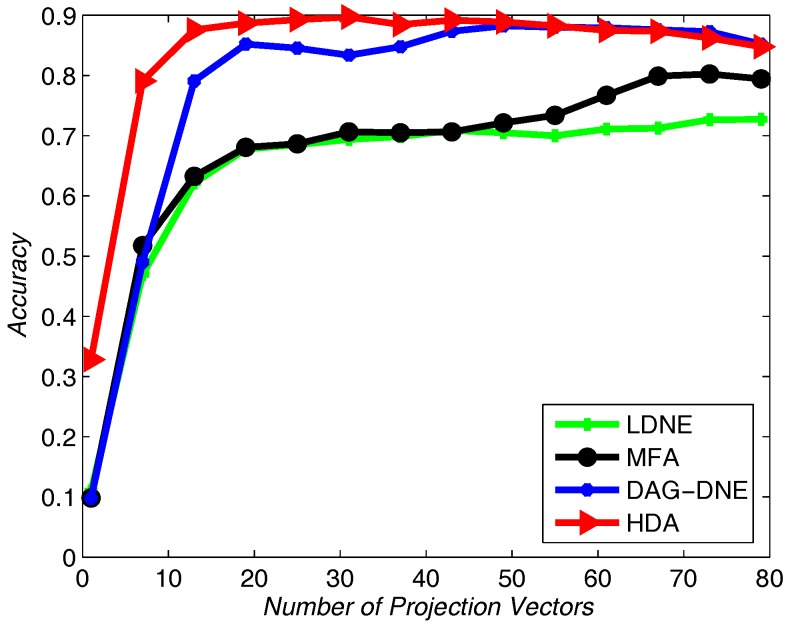
Accuracy vs. Number of Projection Vectors on the UMIST dataset under K=1.

**Figure 8:**
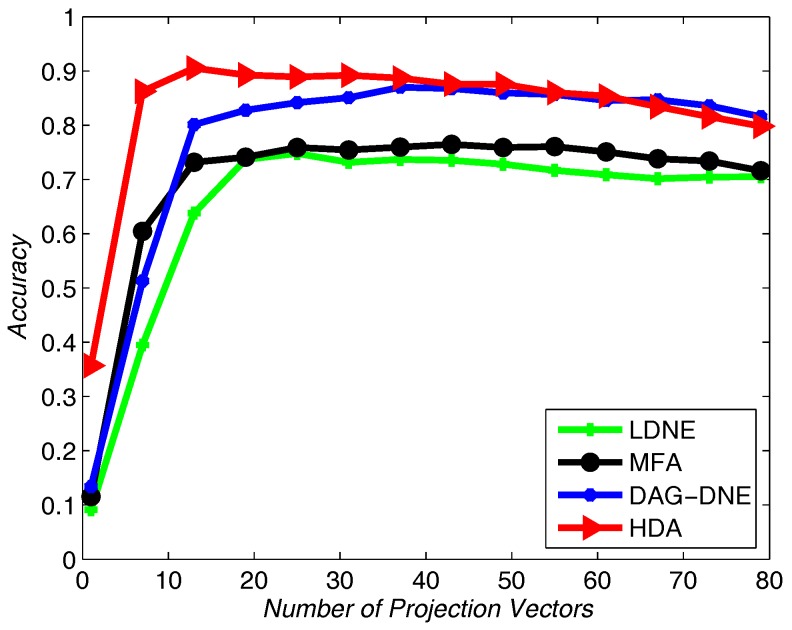
Accuracy vs. Number of Projection Vectors on the UMIST dataset under K=3.

**Figure 9:**
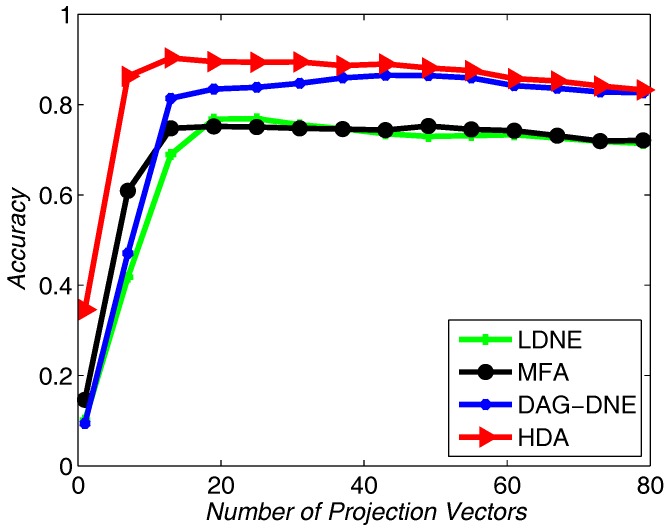
Accuracy vs. Number of Projection Vectors on the UMIST dataset under K=5.

Similar to applying the Yale dataset, we use UMIST dataset to test the recognition rate of the proposed algorithm and the other three algorithms. We select 20% from the UMIST dataset as training samples and use others as testing samples. Figure 7, Figure 8 and Figure 9 show the average accuracy rate of different *K* after running the four algorithms 10 times. As shown in these figures, our algorithm reaches the top and presents the best recognition accuracy compare to the other three algorithms.

### 4.3. ORL Dataset

The Olivetti Research Lab dataset is composed of 40 distinct objects. Each of them contains 10 different images. The ORL dataset contains images in different conditions, such us light conditions, facial expressions, etc. All of the images were taken under a dark homogenous background in an upright and frontal position. The size of each image is 112 × 92 pixels, with 256 gray levels per pixel. Similarly, we resize those image to 32 × 32.

**Figure 10:**
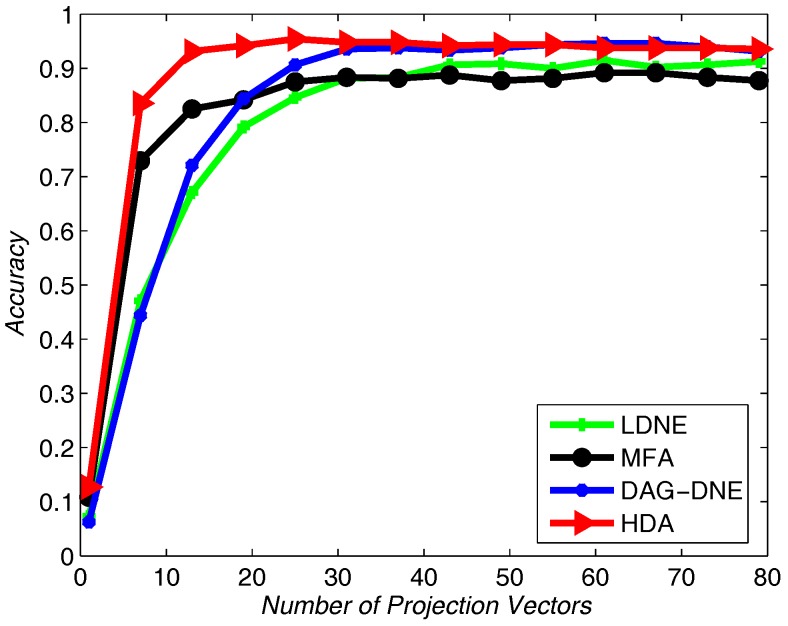
Accuracy vs. Number of Projection Vectors on the Olivetti Research Lab (ORL) dataset under K=1.

**Figure 11:**
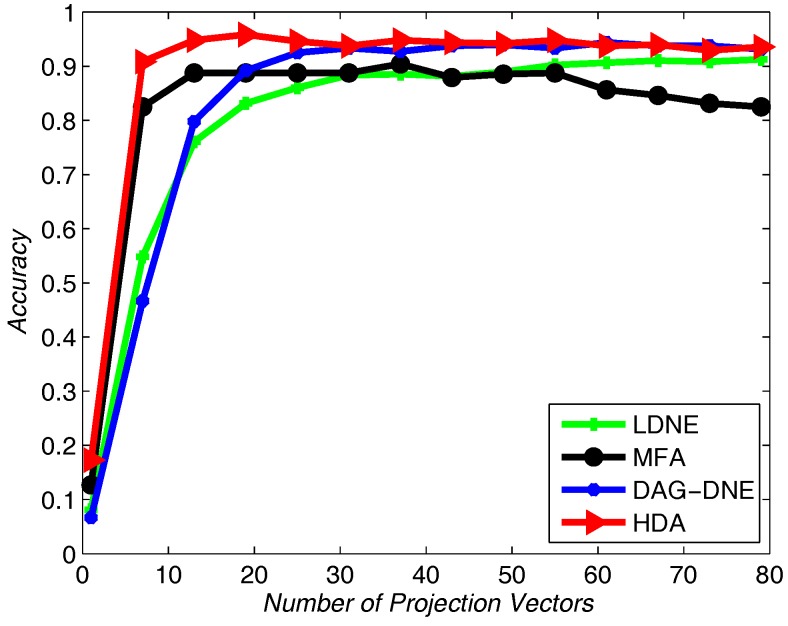
Accuracy vs. Number of Projection Vectors on the ORL dataset under K=3.

**Figure 12:**
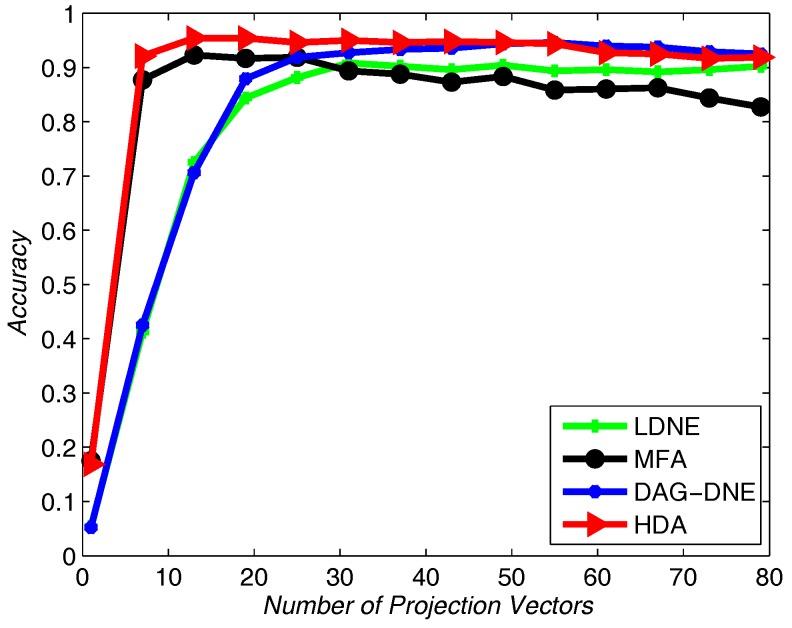
Accuracy vs. Number of Projection Vectors on the ORL dataset under K=5.

Similarly, we choose the nearest neighbor parameter *K* be 1, 3 and 5. Take 60% training samples from the original dataset, and the other 40% as testing samples. As shown in Figure 10, Figure 11 and Figure 12, HDA presents the best accuracy rate compared to the other algorithms after 10 runs and HDA is almost the best one under a different number of projection vectors.

Table 1 shows the comparison of average recognition accuracy for different algorithms and different *K* values. HDA has higher recognition accuracy than the other three algorithms. It improves the classification performance through a dimensional reduction method.

## 5. Conclusions

The collection of a huge amount of high-dimensional sensor intelligent data from the Internet of Things (IoT) causes an information overload problem, so the importance of this research is even more prominent. In this paper, a novel algorithm named hierarchical discriminant analysis is proposed, which aims to find a good latent subspace and preserves the intrinsic structure of the high-dimensional intelligent data. Our proposed algorithm constructs two adjacency graphs to preserve the local structure and deal with optimization problems separately. The experimental results show that HDA is more effective than MFA, LDNE and DAG-DNE on three real image datasets. In other words, hierarchical discriminant analysis can generate a good discriminant subspace. However, HDA is still a linear algorithm, so future work will focus on extending it to be nonlinear to improve the classification performance in cloud computing environments [17,18,19].

## Figures and Tables

**Table 1:** Performance comparison of the algorithms on three datasets with different numbers of *K*.

Algorithm/Result	Yale			ORL			UMIST		
	K=1	K=3	K=5	K=1	K=3	K=5	K=1	K=3	K=5
8-10 LDNE	0.7733	0.7600	0.7067	0.9208	0.9146	0.9208	0.7369	0.7480	0.7775
MFA	0.7600	0.7378	0.7244	0.8937	0.9042	0.9229	0.8034	0.7701	0.7627
DAG-DNE	0.8000	0.7911	0.7378	0.9500	0.9437	0.9521	0.8869	0.8699	0.8655
HDA	0.8444	0.8400	0.8178	0.9542	0.9708	0.9583	0.8973	0.9106	0.9061
